# Effects of Ammonia Concentration on Sperm Vitality, Motility Rates, and Morphology in Three Marine Bivalve Species: A Comparative Study of the Noble Scallop *Mimachlamys nobilis*, Chinese Pearl Oyster *Pinctada fucata martensii*, and Small Rock Oyster *Saccostrea mordax*

**DOI:** 10.3390/biology13080589

**Published:** 2024-08-03

**Authors:** Minghao Li, Jiong Wu, Rui Yang, Zhengyi Fu, Gang Yu, Zhenhua Ma

**Affiliations:** 1 Key Laboratory of Efficient Utilization and Processing of Marine Fishery Resources of Hainan Province, Sanya Tropical Fisheries Research Institute, Sanya 572018, China; 2 South China Sea Fisheries Research Institute, Chinese Academy of Fishery Sciences, Guangzhou 510300, China; 3 Hainan Engineering Research Center for Deep-Sea Aquaculture and Processing, Sanya 572018, China; 4 International Joint Research Center for Conservation and Application of Fishery Resources in the South China Sea, Sanya 572018, China; 5 College of Fisheries, Tianjin Agricultural University, Tianjin 300384, China; 6 College of Science and Engineering, Flinders University, Adelaide 5001, Australia

**Keywords:** *Mimachlamys nobilis*, *Pinctada fucata martensii*, *Saccostrea mordax*, ammonia water, sperm vitality, motility rate, sperm morphology

## Abstract

**Simple Summary:**

This study explores how different concentrations of ammonium ions affect the sperm health of three important bivalve species: Noble Scallop *Mimachlamys nobilis*, Chinese Pearl Oyster *Pinctada fucata martensii*, and Small Rock Oyster *Saccostrea mordax*. Bivalves are crucial to aquatic ecosystems, and understanding the factors that impact their reproduction is essential. Our research found that higher levels of ammonium reduced sperm vitality and movement in Noble Scallop and Small Rock Oysters, especially at the highest concentration we tested. Interestingly, the Chinese Pearl Oyster showed improved sperm vitality and movement at moderate ammonium levels. Additionally, we observed the increased curling of sperm tails in Noble Scallops and Small Rock Oysters with higher ammonium levels, but this effect was not seen in Chinese Pearl Oysters. These findings highlight how varying environmental conditions can differently affect species and emphasize the importance of maintaining balanced ammonium levels in aquatic habitats. This knowledge is valuable for protecting the reproductive health of these bivalves, which play a key role in the health and sustainability of aquatic ecosystems.

**Abstract:**

Ammonium (NH_4_^+^) plays a crucial role in the reproductive processes of key biotic groups in aquatic ecosystems—bivalves. This study aims to elucidate the effects of three different ammonium ion concentrations on sperm vitality, swimming kinematics, and morphology of *Mimachlamys nobilis*, *Pinctada fucata martensii*, and *Saccostrea mordax*. The results indicate that the sperm vitality and motility rates of *M.nobilis* and *S. mordax* are inversely proportional to the ammonium concentration, especially in the treatment group with an ammonium concentration of 3 mmol/L, where the decrease in sperm vitality and motility is most significant. In contrast, the sperm of *P. fucata martensii* reacted differently to increasing ammonium concentrations. After the addition of 2 mmol/L of ammonium, the sperm vitality and motility of *P. fucata martensii* reached a peak, showing a significant stimulatory effect. Additionally, as the ammonium concentration increased, the curling of the sperm flagella in *M.nobilis* and *S. mordax* increased. However, sperm flagella curling in *P. fucata martensii* showed no change compared to the control group. This study provides insights into the effects of ammonium concentrations on the sperm vitality and motility of three marine bivalve species and highlights the importance of sperm flagella curling as a factor affecting sperm.

## 1. Introduction

Mollusks represent the second-largest animal phylum, comprising nearly 100,000 species, of which 15,000 are bivalves [1]. Shellfish farming is considered a sustainable method of mariculture, as most bivalves reproduce through external fertilization. Male and female individuals release sperm and eggs into the water, where fertilization occurs [2]. The activity of sperm in different marine bivalves exhibits specific characteristics, such as sperm from *M. nobiliss*, activated by natural seawater, which can maintain high-speed swimming for at least 40 h, characterized as “marathon-type sperm” [3,4]. While the *P. fucata martensii* sperm are categorized as “sprint-type sperm,” exhibiting high motility shortly after activation but for a brief duration, research indicates that sperm obtained from artificially dissected *P. fucata martensii* do not move in natural seawater and lack fertilization capabilities. Activation requires specific chemicals, which maintain vitality for about 24 h [5,6]. Additionally, it has been reported that *S. mordax* sperm can maintain over 60% of their vitality for up to 24 h in natural seawater [7,8]. These three types of shellfish are all marine bivalves and are widely distributed in the tropical waters of the South China Sea. They have high economic value, yet, there is currently limited research on the different activation mechanisms of sperm for these three types of shellfish [9,10,11,12,13].

The differences in the concentration of various related ionic components in the aquatic environment and marine animal sperm may be a source of sperm motility activation. The impact of different types of ions on sperm vitality varies; cations generally play a major role in the activation process of sperm, while the role of anions is relatively minor [14]. The influence of ions on sperm vitality is often closely related to the external environment [15]. Among these, the NH_4_^+^ ion, as one of the natural seawater cations, may affect the function of intracellular Na^+^, K^+^, and Ca_2_^+^ ion channels, which are crucial for the motility and functionality of sperm [16]. Additionally, NH_4_^+^ ions can react with water to produce H+ and NH_3_^+^ ions, thereby affecting the acid-base balance of the water. Sperm need to maintain their activity and functionality in a relatively stable pH environment [17]. It has been reported that NH_4_^+^ ions disrupt multiple signaling pathways, including reducing the activity of Na/K ATPase and the levels of protein kinase B (AKT), leading to a decline in boar sperm vitality [18]. Studies have shown that the acrosome reaction necessary for the movement of sea urchin (*Arbacia punctulata*) sperm requires a continuous increase in intracellular Ca_2_^+^ and NH_4_^+^ [19]. It is worth noting that ammonia concentrations in marine environments are influenced by a variety of natural and anthropogenic factors. Ammonia from natural sources mainly originates from microbial metabolism, and environmental factors such as water temperature, pH, and salinity also affect its concentration and form [20]. It has been reported that ammonia concentrations in natural environments are typically very low, ranging from approximately 0.01 to 0.5 µmol/L, although this concentration can vary between different marine areas and seasons [21]. With increasing global attention on climate change, the maritime industry is exploring the use of ammonia as a new decarbonization fuel. Ammonia is an efficient energy carrier and has the potential for zero carbon emissions, but it may enter the marine environment during its production, storage, and use, thereby increasing ammonia concentrations in seawater [22]. Given the impact of ammonia on aquatic organisms, particularly its physiological effects on shellfish, understanding the impact of changes in ammonia concentrations on their reproductive capacity is crucial. Research suggests that even slight changes in ammonia concentration can affect sperm motility and fertilization rates in shellfish [23]. Therefore, the mechanisms related to the differential impact of NH_4_^+^ on sperm across different species still require further investigation. 

The overall motility of a sperm population is referred to as sperm vitality, which is an important parameter for assessing the quality of animal semen. This includes metrics such as the motility rate (the proportion of motile sperm to the total number of sperm), the intensity of sperm movement, the duration of movement, and the speed of movement [24]. Sperm vitality refers to the overall movement behavior of a sperm population and is a critical parameter for assessing the quality of animal semen. Sperm-swimming kinematics, including curvilinear velocity, average path velocity, and straight-line velocity, are important parameters for assessing sperm motility [25]. These metrics not only provide quantitative information on sperm movement efficiency but also reveal sperm adaptability in various chemical and physical environments [26]. The research found that the swimming kinematic characteristics of black-lip pearl oyster (*Pinctada margaritifera var: cumingii*) sperm, such as velocity and vitality, are significantly associated with morphology and fertilization success rates [27]. It has been reported that environmental factors influence the duration of activity in Greenshell^Tm^ mussel (*Perna canaliculus*) sperm, and this duration is proportional to fertilization rates [28]. Additionally, morphological attributes of sperm, such as the head size and tail length, are important indicators for assessing their quality and fertilization capabilities. Sperm with morphological abnormalities often have a reduced fertilization ability, which is closely related to their motility and the integrity of their genetic material [29]. Research has shown that samples with a higher proportion of morphologically normal sperm usually exhibit a higher fertilization success rate based on observations of the morphology and ultrastructure of European flat oyster (*Ostrea edulis*) sperm [30]. It is noteworthy that ammonia concentrations may affect oocytes and, consequently, fertilization. In environments with high ammonia concentrations, oocytes may undergo various physiological and biochemical changes that could reduce their likelihood of fertilization or affect post-fertilization embryonic development [31]. Specifically, ammonia can affect oocytes through multiple mechanisms. Studies have shown that ammonia can directly exert toxic effects on oocytes, impacting their viability [32]. It has been reported that ammonia may disrupt normal cellular metabolism, leading to impaired energy production, which is crucial for oocyte maturation and subsequent fertilization processes [33]. Additionally, ammonia increases the production of reactive oxygen species (ROS) within cells, potentially causing oxidative stress that can damage cellular structures and functions [34]. Research has confirmed that ammonia may interfere with receptors on the oocyte surface or other molecules related to fertilization, affecting the recognition and binding processes between sperm and egg [35]. Further exploration into the mechanisms by which ammonia concentrations impact oocytes and fertilization is warranted.

This research aims to explore the effects of NH_4_^+^ on sperm vitality, swimming kinematics, and the morphology of three marine bivalve species—the *M. nobiliss*, *P. fucata martensii*, and *S. mordax*. It seeks to reveal the physiological state of sperm across different marine bivalve species and the impact of environmental factor variations on sperm. By comprehensively examining these indicators, this research hopes to provide a deeper understanding of the reproductive biology of marine organisms while offering scientific rationale and technical support for marine aquaculture and conservation.

## 2. Materials and Methods

### 2.1. Materials

The *M. nobiliss*, *P. fucata martensii*, and *S. mordax* used in the experiment were provided by the Tropical Aquaculture Research and Development Center of the South China Sea Fisheries Research Institute, Chinese Academy of Fishery Sciences, located in Xincun Port, Lingshui Li Autonomous County, Hainan Province. Individuals of one-year-old sexual maturity were selected for *M. nobiliss*, *P. fucata martensii*, and *S. mordax*. During the sperm collection period, the experimental animals were temporarily kept in seawater with a salinity of (35 ± 0.2)‰, a temperature of (25 ± 2) °C, and a pH of 8.0 ± 0.2 for standby. 

### 2.2. Trait Data Collection

We selected 70 individuals each of *M. nobiliss*, *P. fucata martensii*, and *S. mordax*. From there, we chose the three male individuals with the fullest gonads and highest maturity for each species. We then measured and recorded the shell length, shell height, shell width, wet weight, soft body weight, and gonad weight for these selected individuals. The results are presented in (Table 1).

### 2.3. Sperm Collection

Seventy individuals each of *M. nobiliss*, *P. fucata martensii*, and *S. mordax* were selected, with the three male parental individuals exhibiting the fullest gonads and highest maturity chosen from each species. A sterile scalpel was used to cut open one side of the adductor muscle of the experimental animals, exposing the visceral mass with gonads covering almost the entire visceral sac, appearing white or pale yellow. The gonads were then dissected, thoroughly dried with clean absorbent paper to remove any mixture of seawater and tissue fluid, and were weighed. 

The obtained gonads of the experimental animals were placed on a 500-mesh silk screen, thoroughly minced with scissors, and rinsed with natural seawater to remove excess tissue fragments. This process yielded relatively clean diluted sperm suspensions of *M. nobiliss*, *P. fucata martensii*, and *S. mordax*, diluted to 2 times their original concentration. The suspensions were then placed in sterilized beakers and stored at 4 °C for later use.

### 2.4. Experimental Setup with Different Concentrations of Ammonia Solution on Sperm

In total, 1 mL of sperm suspension from *M. nobiliss*, *P. fucata martensii*, and *S. mordax* was taken and diluted with 9 mL of artificial seawater at 4 °C, placed into 15 mL conical plastic centrifuge tubes, serving as the control groups. Similarly, 1 mL of the sperm suspension from each species was mixed with 9 mL of artificial seawater containing different concentrations of ammonium ions (1 mmol/L, 2 mmol/L, 3 mmol/L). These concentrations were achieved by diluting 68.12 µL, 150 µL, and 204.36 µL of a 25% ammonia solution with natural seawater to 1 L, which was thoroughly mixed and prepared fresh for use. The 25% ammonia solution used in the experiments was produced by Xilong Scientific Co., Ltd., Shantou, China, with a purity of 99% for the analytical reagent. The choice of ammonia concentration in the experiment was based on the results of multiple preliminary experiments and was selected as the optimal concentration range tailored to the characteristics of the experimental species. These samples were also placed into 15 mL conical plastic centrifuge tubes and configured as experimental groups. The pH of all experimental samples was measured and recorded using a pH pen meter (PH-pro series pH pen meter produced by Lichen Instruments Technology Co., Ltd., Shanghai, China). Each group had 3 replicates (Table 2). 

### 2.5. The Impact of Different Concentrations of Ammonia Water on the Vitality of Sperm from M. nobiliss, P. fucata martensii, and S. mordax

At 9 time points (0, 4, 8, 12, 16, 20, 24, 32, and 40 h), 5 μL samples were taken and smeared in a sperm counting chamber for observation and the recording of sperm vitality under a sperm quality analyzer. Each group was tested in triplicate.

### 2.6. The Impact of Different Concentrations of Ammonia Water on Curvilinear Velocity, Average Path Velocity, and Straight-Line Velocity of Sperm from M. nobiliss, P. fucata martensii, and S. mordax

At 9 time points (0, 4, 8, 12, 16, 20, 24, 32, and 40 h), 5 μL samples were analyzed in a sperm counting chamber under a sperm quality analyzer to observe and record the curvilinear velocity, average path velocity, and straight-line velocity of the sperm. Each condition was tested in triplicate.

### 2.7. Observation of Sperm Morphology under Different Concentrations of Ammonia Water

Based on the impact of different concentrations of ammonia water on the vitality, curvilinear velocity, average path velocity, and straight-line velocity of sperm from *M. nobiliss*, *P. fucata martensii*, and *S. mordax*, 1 mL of sperm samples with and without added ammonia water was fixed with a solution of 4% paraformaldehyde and 1% glutaraldehyde (comprising 10 mL of 50% glutaraldehyde, 20 g paraformaldehyde, and 3.029 g Tris in 500 mL of filtered seawater). Following gradient dehydration with alcohol and the addition of isoamyl acetate, the samples were dried using a critical point dryer (LEICA EM CPD 300, produced by Leica Microsystems, Beijing, China) and sputter-coated with gold in an IB-5 ion sputtering device (Eiko IB5 Ion Coater, produced by EIKO Kogyo Co., Ltd., Tokyo, Japan). Sperm morphology was then observed using a scanning electron microscope (Beionmed ProX G6 model, produced by Beionmed Pharmaceutical Technology Co., Shanghai, China).

### 2.8. Quality Evaluation of Sperm Vitality

Observations and records were made at nine consistent time points. The sperm of *M. nobiliss*, *P. fucata martensii*, and *S. mordax* were analyzed using a sperm quality analyzer (Beionmed S3, produced by Beionmed, China), capturing 400 to 600 sperm per lens. Six fields of view were randomly selected for each sample to assess sperm vitality. The parameters used for evaluating sperm vitality quality included sperm vitality (progressive motility + non-progressive motility, %), curvilinear velocity (VCL), average path velocity (VAP), and straight-line velocity (VSL). 

### 2.9. Data Analysis

The data of sperm parameters measured by the Computer-Assisted Sperm Analysis (CASA) system during the experiment were statistically analyzed using SPSS 27.0. Before performing the ANOVA, the data underwent arcsine transformation to meet the requirements for normal distribution. Additionally, distribution analyses were performed on the data to verify the normality and homogeneity of variances, ensuring the applicability and validity of the variance analysis. All related statistical hypothesis tests were satisfied; thus, the results of the ANOVA are reliable. One-way Analysis of Variance (ANOVA) was conducted with the software, where *p* < 0.05 indicated a significant difference, *p* < 0.01 indicated a highly significant difference, and *p* < 0.001 indicated an extremely significant difference. Results are presented as the mean ± standard deviation (mean ± S.D). Graphs were created using Origin 2021. 

## 3. Results

### 3.1. Impact of Different Ammonia Concentrations on the Vitality of Sperm from M. nobiliss, P. fucata martensii, and S. mordax

**Figure 1:**
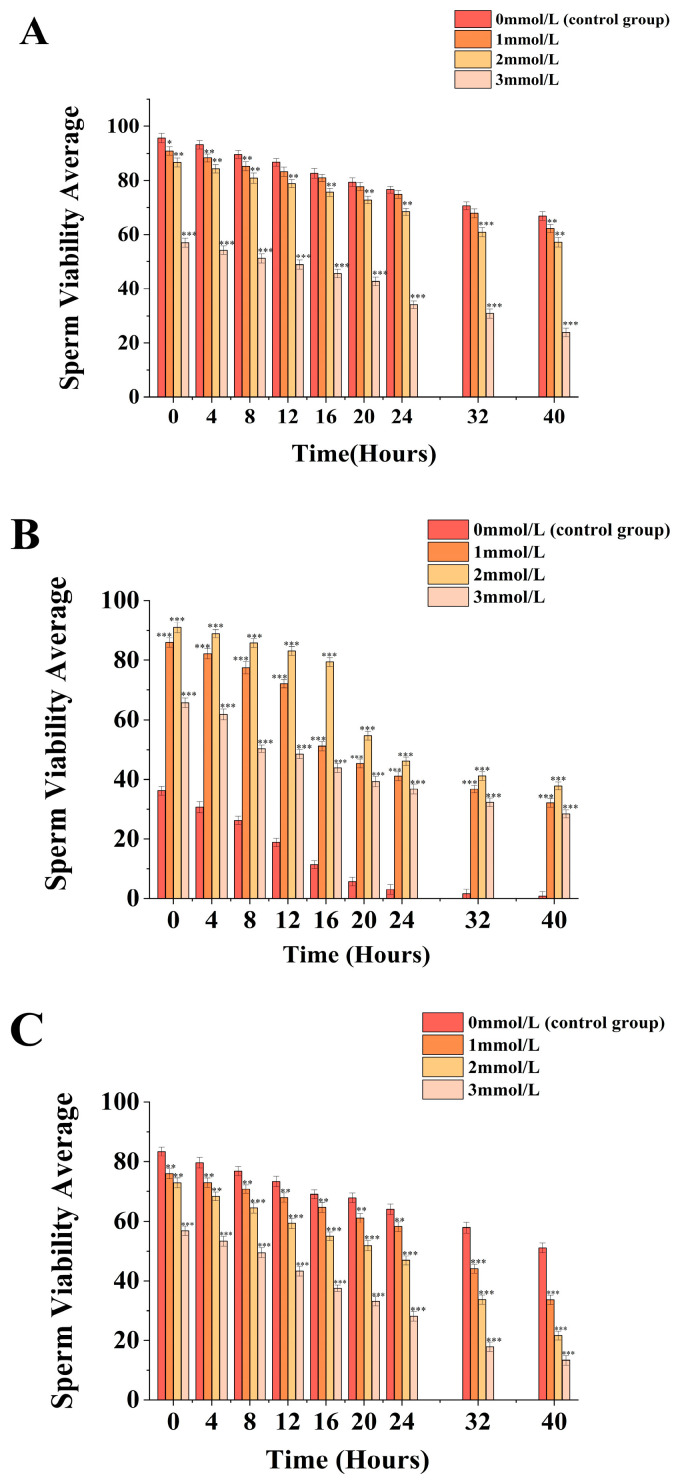
Impact of different ammonia concentrations (0, 1, 2, and 3 mmol/L) on sperm viability (µ) after 40 h of exposure in *Mimachlamys nobilis* (**A**), *Pinctata fucata martensii* (**B**) and *Saccostrea mordax* (**C**). (* *p <* 0.05, ** *p <* 0.01, *** *p <* 0.001). X-axis = sperm viability.

Under the condition of whether ammonia water was added or not (Figure 1A), sperm vitality was inversely proportional to the concentration of ammonia water. For the *M. nobilis* without ammonia water treatment, sperm vitality continuously decreased from (95.67 ± 1.74)% at 0 h to (66.93 ± 1.57)% at 40 h. For the 1 mmol/L and 2 mmol/L ammonia water treatment groups, sperm vitality decreased from (90.93 ± 1.55)% and (86.61 ± 1.61)% to (62.2 ± 1.48)% and (57.4 ± 1.75)%, respectively. Moreover, the difference in sperm motility between the 1 mmol/L ammonia water treatment group and the control group was within 10%, with significantly lower sperm vitality at 0 h (*p <* 0.05) and an even more significantly lower vitality at 4 h, 8 h, and 40 h (*p <* 0.01) compared to the control group. Additionally, the sperm vitality of the 3 mmol/L ammonia water treatment group at 0 h was (57.03 ± 1.67)%, which then decreased from (57.03 ± 1.67)% to (23.87 ± 1.57)%, with a reduction of more than 58%. At each time point, it showed the most significant decrease compared to the control group (*p <* 0.001).

Under the condition of whether ammonia water was added or not (Figure 1B), the sperm vitality of untreated *P. fucata martensii* decreased from (36.17 ± 1.55)% at 0 h to (0.81 ± 1.47)% at 40 h. The sperm vitality in the 1mmol/L, 2mmol/L, and 3mmol/L ammonia water treatment groups decreased from (85.97 ± 1.57)%, (90.97 ± 1.75)%, and (65.7 ± 1.6)% to (32.13 ± 1.45)%, (37.85 ± 1.38)%, and (28.47 ± 1.31)%, respectively. The sperm vitality in the 1mmol/L, 2mmol/L, and 3mmol/L ammonia water treatment groups showed the largest average decrease during the time periods of 12–16 h, 16–20 h, and 4–8 h, with decreases of (20.97 ± 3.74)%, (24.69 ± 3.92)%, and (11.51 ± 2.68)%, respectively, representing the most significant declines compared to other time periods (*p <* 0.001). By 40 h, the sperm vitality in the 1mmol/L, 2mmol/L, and 3mmol/L ammonia water treatment groups was significantly higher than the control group (*p <* 0.001).

Under the condition of whether ammonia water was added or not (Figure 1C), sperm vitality was inversely proportional to the ammonia concentration. For *S. mordax*, sperm vitality continuously decreased from (83.43 ± 1.47)% at 0 h to (51.1 ± 1.61)% at 40 h. For the 1 mmol/L and 2 mmol/L ammonia water treatment groups, sperm vitality decreased from (75.97 ± 1.62)% and (72.97 ± 1.61)% to (33.63 ± 1.59)% and (21.63 ± 1.49)%, respectively. The 1 mmol/L ammonia water treatment group showed a significant decrease in sperm vitality compared to the control group at 40 h (*p <* 0.001) within a 34% margin, with sperm vitality appearing significantly lower from 0 to 24 h (*p <* 0.01) and extremely significantly lower from 32 to 40 h (*p <* 0.001) compared to the control group. The 2 mmol/L ammonia water treatment group showed the largest difference in sperm vitality compared to the control group at 40 h (*p <* 0.001), within a 58% margin, with sperm vitality appearing significantly lower throughout the 0 to 40 h period compared to the control group (*p <* 0.001). Additionally, the sperm vitality of the 3 mmol/L ammonia water treatment group at 0 h was (56.77 ± 1.54)%, which then decreased to (13.36 ± 1.65)% at 40 h, with a reduction of more than 76%. At each time point, it showed a significant decrease compared to the control group (*p* < 0.001).

### 3.2. The Effects of Different Concentrations of Ammonia Water on the Curvilinear Velocity of Sperm from M. nobiliss, P. fucata Martensii, and S. mordax

**Figure 2:**
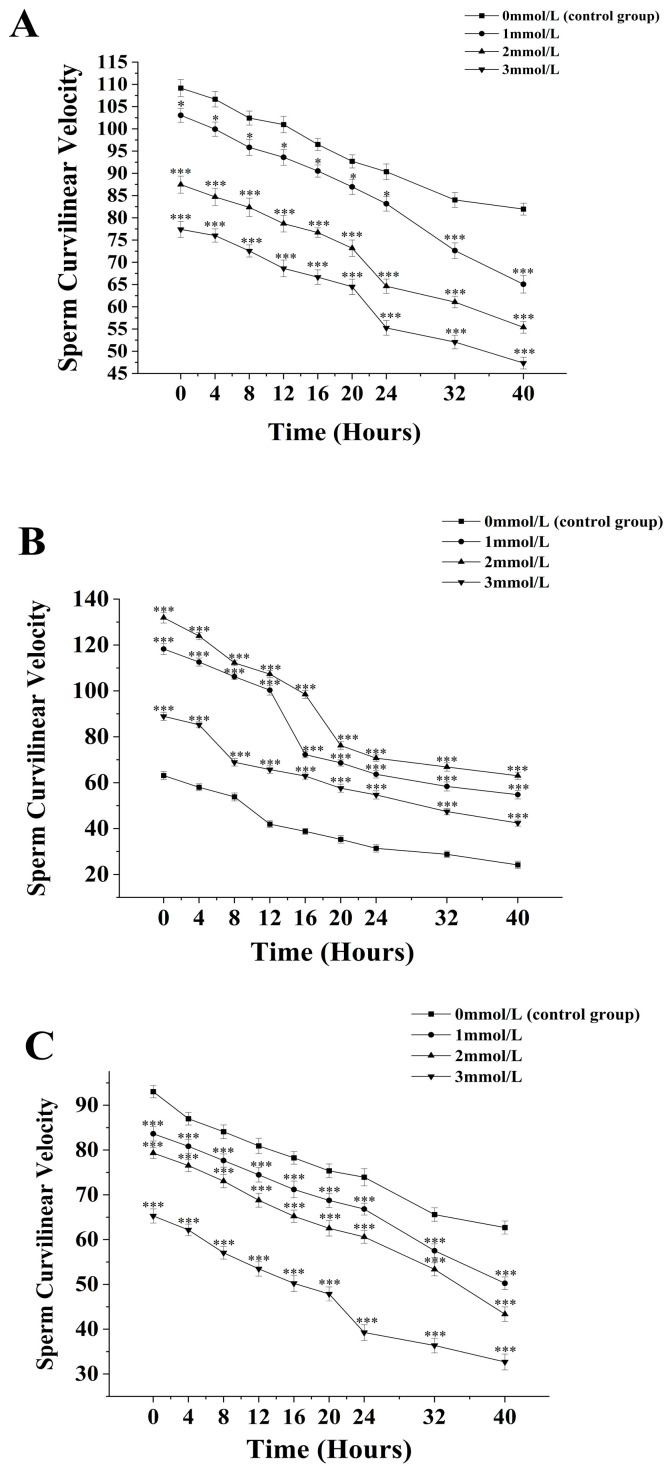
Effect of different ammonia concentrations (0, 1, 2, and 3 mmol/L) on sperm curvilinear velocity (µ) after 40 h of exposure in *Mimachlamys nobilis* (**A**), *Pinctata fucata martensii* (**B**) and *Saccostrea mordax* (**C**). (* *p <* 0.05, *** *p <* 0.001). X-axis = sperm curvilinear velocity.

The curvilinear velocity of sperm from *M. nobilis* is inversely proportional to the concentration of ammonia solution (Figure 2A). In the group without added ammonia, the sperm curvilinear velocity decreased from (109.17 ± 1.93) µm/s at 0 h to (81.97 ± 1.35) µm/s at 40 h. In the 1 mmol/L treatment group, the velocity decreased from (103.06 ± 1.57) µm/s at 0 h to (65.07 ± 1.97) µm/s at 40 h, with a significant drop after 32 h (*p <* 0.05). In the 2 mmol/L group, the velocity dropped from (87.47 ± 1.91) µm/s at 0 h to (55.4 ± 1.32) µm/s at 40 h, with a significant decrease noted at 24 h (*p <* 0.05). The 3 mmol/L group showed a decrease from (77.4 ± 1.82) µm/s at 0 h to (55.4 ± 1.32) µm/s at 40 h, with a significant drop at 24 h (*p <* 0.05).

For *P. fucata martensii* (Figure 2B), the curvilinear velocity of sperm without ammonia was significantly lower than the groups treated with ammonia at all time points (*p <* 0.001). The untreated group’s velocity decreased from (63.13 ± 1.8) µm/s at 0 h to (24.2 ± 1.59) µm/s at 40 h. The 1 mmol/L group saw a decrease from (118.27 ± 2.36) µm/s at 0 h to (54.77 ± 1.82) µm/s at 40 h, with a significant drop at 20 h (*p <* 0.001). The 2 mmol/L group decreased from (131.93 ± 2.32) µm/s at 0 h to (63.03 ± 1.57) µm/s at 40 h, also showing a significant drop at 20 h (*p <* 0.001). The 3 mmol/L group experienced a decline from (88.93 ± 1.78) µm/s at 0 h to (42.43 ± 1.28) µm/s at 40 h, with a significant drop at 8 h (*p <* 0.001).

The curvilinear velocity of sperm from *S. mordax* was inversely proportional to the ammonia concentration (Figure 2C). The velocity in the control group decreased from (93.04 ± 1.35) µm/s at 0 h to (62.7 ± 1.46) µm/s at 40 h. In the 1 mmol/L group, the velocity dropped from (83.63 ± 1.59) µm/s at 0 h to (50.27 ± 1.41) µm/s at 40 h, with significant declines noted at 32 h and 40 h (*p <* 0.05). The 2 mmol/L group decreased from (79.35 ± 1.25) µm/s at 0 h to (43.63 ± 1.62) µm/s at 40 h with significant decreases at 24 h and 40 h (*p <* 0.05). The 3 mmol/L group showed a decline from (65.3 ± 1.59) µm/s at 0 h to (32.7 ± 1.76) µm/s at 40 h, with a significant drop at 24 h (*p <* 0.05).

### 3.3. The Effects of Different Concentrations of Ammonia Water on the Average Path Velocity of Sperm from M. nobiliss, P. fucata Martensii, and S. mordax

**Figure 3:**
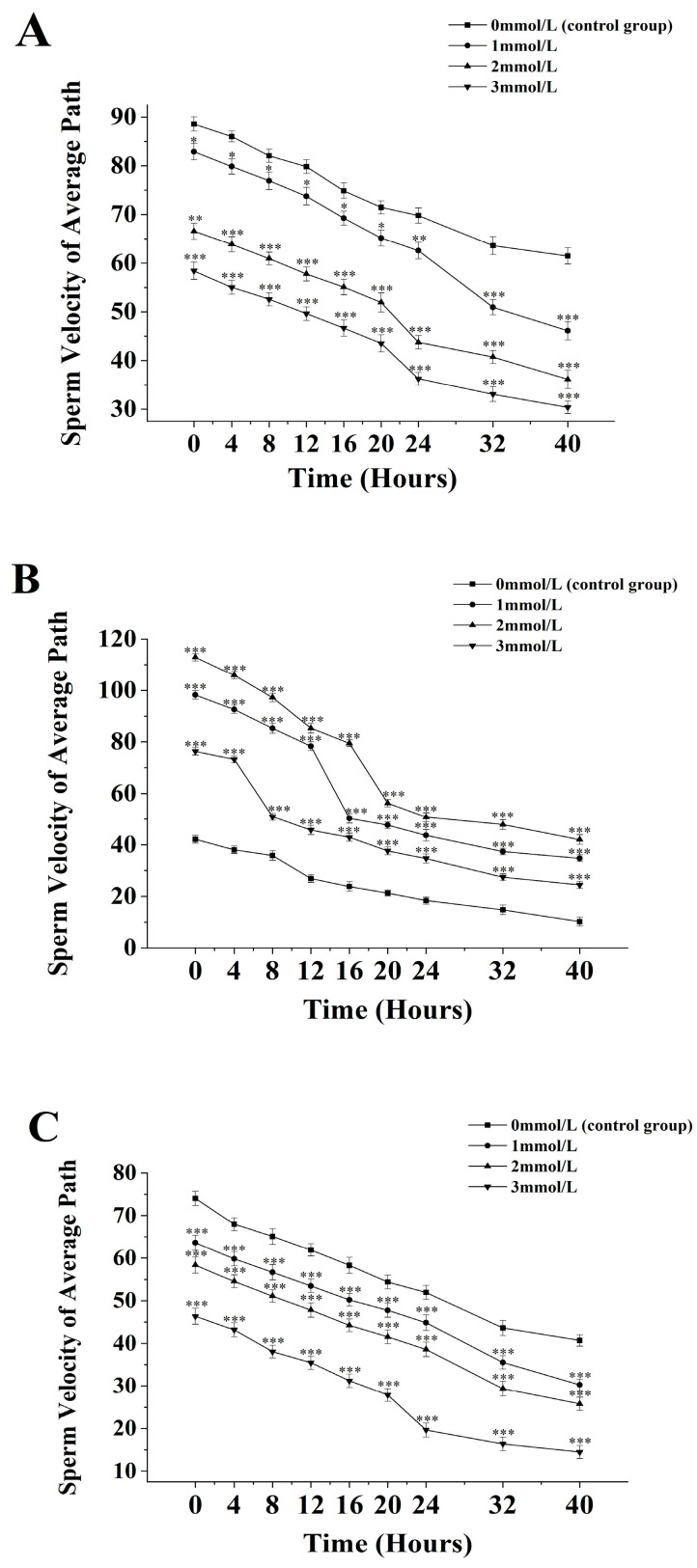
Effect of different ammonia concentrations (0, 1, 2, and 3 mmol/L) on sperm path velocity (µ) after 40 h of exposure in *Mimachlamys nobilis* (**A**), *Pinctata fucata martensii* (**B**), and *Saccostrea mordax* (**C**). (* *p <* 0.05, ** *p <* 0.01, *** *p <* 0.001). X-axis = sperm path velocity.

The average path velocity of sperm from *M. nobilis* is inversely proportional to the concentration of ammonia solution (Figure 3A). In the group without ammonia, the sperm’s average path velocity decreased from (88.57 ± 1.47) μm/s at 0 h to (61.47 ± 1.67) μm/s at 40 h. In the 1 mmol/L treatment group, the velocity decreased from (82.9 ± 1.69) μm/s at 0 h to (46.13 ± 1.86) μm/s at 40 h, with a significant drop at 32 h (*p <* 0.05). In the 2 mmol/L group, the velocity dropped from (66.57 ± 1.65) μm/s at 0 h to (36.13 ± 1.86) μm/s at 40 h, with a significant decrease at 24 h (*p <* 0.05). The 3 mmol/L group showed a decrease from (58.4 ± 1.75) μm/s at 0 h to (30.35 ± 1.31) μm/s at 40 h, with a significant drop after 24 h (*p <* 0.05).

For *P. fucata martensii* (Figure 3B), the average path velocity of sperm without ammonia was significantly lower than in groups treated with ammonia at all time points (*p <* 0.001). The untreated group’s velocity decreased from (42.13 ± 1.53) µm/s at 0 h to (10.2 ± 1.74) µm/s at 40 h. In the 1 mmol/L group, the velocity decreased from (98.27 ± 1.62) µm/s at 0 h to (34.77 ± 1.32) µm/s at 40 h, with a significant drop at 20 h (*p <* 0.001). In the 2 mmol/L group, the velocity dropped from (112.93 ± 1.44) µm/s at 0 h to (42.03 ± 1.87) µm/s at 40 h, with an additional significant drop at 20 h (*p <* 0.001). The 3 mmol/L group experienced a decline from (76.35 ± 1.47) µm/s at 0 h to (24.43 ± 1.38) µm/s at 40 h, with a significant drop at 8 h (*p <* 0.001).

The average path velocity of *S. mordax* sperm is inversely proportional to the concentration of ammonia solution (Figure 3C). In the control group, the average path velocity decreased from (74.04 ± 1.67) µm/s at 0 h to (40.7 ± 1.36) µm/s at 40 h. In the 1 mmol/L group, the velocity dropped from (63.72 ± 1.59) µm/s at 0 h to (30.16 ± 1.36) µm/s at 40 h, with significant decreases noted at 32 h and 40 h (*p <* 0.05). The 2 mmol/L group decreased from (58.35 ± 1.87) µm/s at 0 h to (26.34 ± 1.54) µm/s at 40 h, with significant decreases after 24 h and 40 h (*p <* 0.05). The 3 mmol/L group showed a decline from (46.35 ± 1.86) µm/s at 0 h to (14.48 ± 1.53) µm/s at 40 h, with a significant drop after 24 h (*p <* 0.05).

### 3.4. The Effects of Different Concentrations of Ammonia Water on the Straight-Line Velocity of Sperm from M. nobiliss, P. fucata martensii, and S. mordax

**Figure 4:**
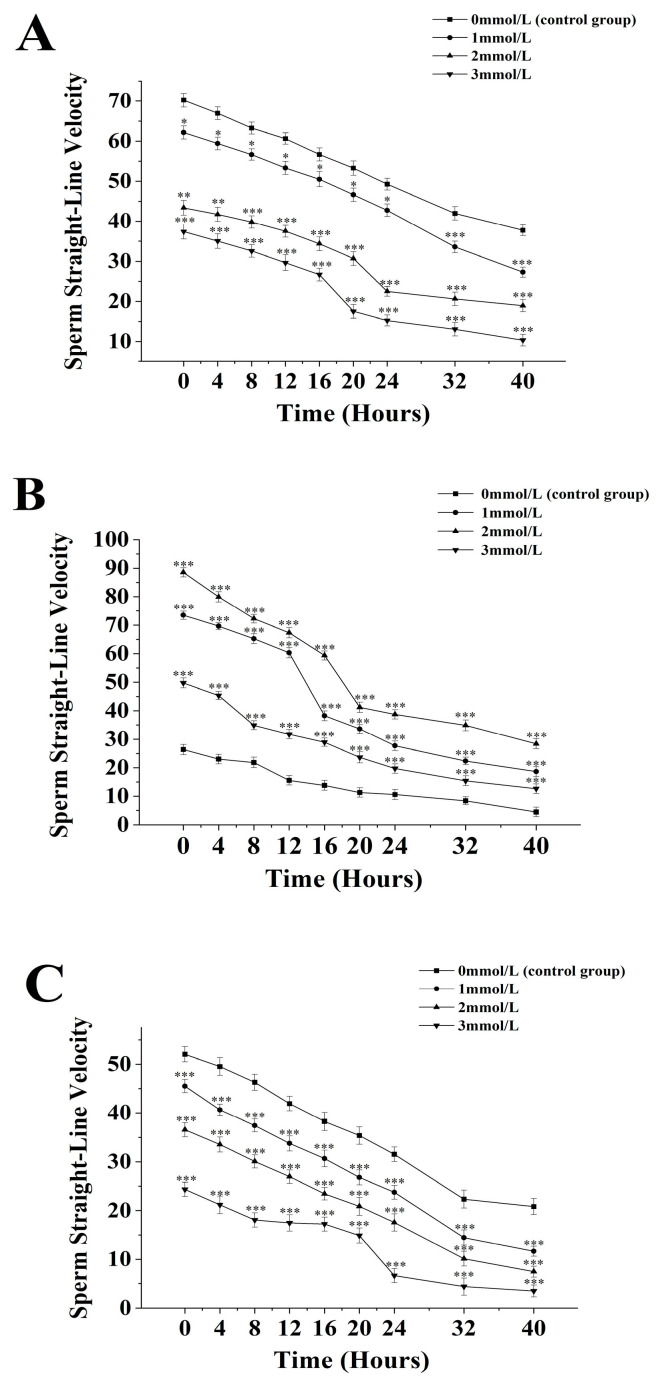
Effect of different ammonia concentrations (0, 1, 2, and 3 mmol/L) on sperm straight-line velocity (µ) after 40 h of exposure in *Mimachlamys nobilis* (**A**) *Pinctata fucata martensii* (**B**), and *Saccostrea mordax* (**C**). (* *p <* 0.05, ** *p <* 0.01, *** *p <* 0.001). X-axis = sperm straight-line velocity.

The straight-line velocity of sperm from *M. nobilis* is inversely proportional to the concentration of ammonia solution (Figure 4A). In the group without ammonia, the sperm’s straight-line velocity decreased from (70.23 ± 1.73) µm/s at 0 h to (37.75 ± 1.36) µm/s at 40 h. In the 1 mmol/L treatment group, the velocity decreased from (62.13 ± 1.63) µm/s at 0 h to (27.27 ± 1.27) µm/s at 40 h, with a significant drop at 32 h (*p <* 0.05). In the 2 mmol/L group, the velocity dropped from (43.38 ± 1.83) µm/s at 0 h to (18.92 ± 1.54) µm/s at 40 h, with a significant decrease at 24 h (*p <* 0.05). In the 3 mmol/L group, the velocity decreased from (37.4 ± 1.86) µm/s at 0 h to (10.35 ± 1.47) µm/s at 40 h, with a significant drop after 20 h (*p <* 0.05).

For *P. fucata martensii* (Figure 4B), the straight-line velocity of sperm without ammonia was significantly lower than in groups treated with ammonia at all time points (*p <* 0.001). The untreated group’s velocity decreased from (42.13 ± 1.53) µm/s at 0 h to (10.2 ± 1.74) µm/s at 40 h. In the 1 mmol/L group, the velocity decreased from (98.27 ± 1.62) µm/s at 0 h to (18.63 ± 1.74) µm/s at 40 h, with a significant drop at 16 h (*p <* 0.001). In the 2 mmol/L group, the velocity dropped from (88.57 ± 1.61) µm/s at 0 h to (28.37 ± 1.79) µm/s at 40 h, with a significant drop at 20 h (*p <* 0.001). The 3 mmol/L group experienced a decline from (49.74 ± 1.73) µm/s at 0 h to (12.64 ± 1.68) µm/s at 40 h, with a significant drop after 8 h (*p <* 0.001).

The straight-line velocity of *S. mordax* sperm is inversely proportional to the concentration of ammonia solution (Figure 4C). In the group without ammonia, the velocity decreased from (52.04 ± 1.55) µm/s at 0 h to (20.82 ± 1.65) µm/s at 40 h. In the 1 mmol/L group, the velocity dropped from (45.54 ± 1.36) µm/s at 0 h to (14.45 ± 1.56) µm/s at 32 h (*p <* 0.05), continuing to decline to (30.16 ± 1.36) µm/s at 40 h. In the 2 mmol/L group, the velocity decreased from (36.57 ± 1.47) µm/s at 0 h to (7.45 ± 1.12) µm/s at 40 h, with significant decreases at 24 h and 40 h (p < 0.05). The 3 mmol/L group showed a decline from (24.35 ± 1.45) µm/s at 0 h to (3.48 ± 1.21) µm/s at 40 h, with a significant drop after 24 h (*p <* 0.05).

### 3.5. Observation of the Effects of Different Concentrations of Ammonia Water on the Morphology of Sperm from M. nobiliss, P. fucata Martensii, and S. mordax with Distinctive Biting or Tooth-like Structures

**Figure 5:**
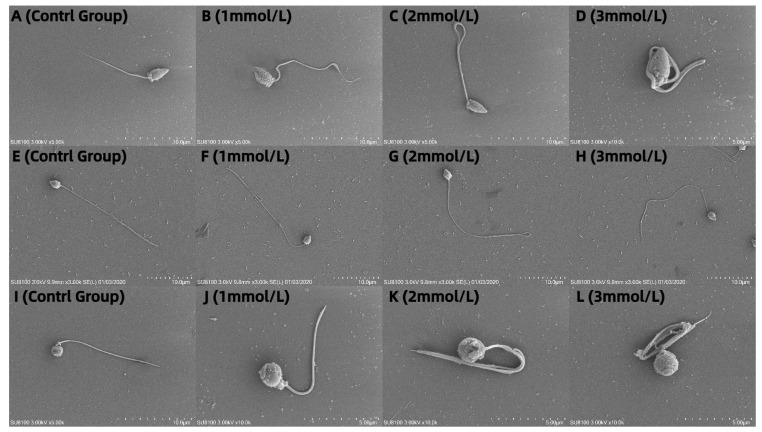
Sperm morphology of three marine bivalve species at different ammonia concentrations: (**A**–**D**) *Mimachlamys nobilis* at 0, 1, 2, and 3 mmol/L; (**E**–**H**) *Pinctata fucata martensii* at 0, 1, 2, and 3 mmol/L, and (**I**–**L**) *Saccostrea mordax* at 0, 1, 2, and 3 mmol/L.

The sperm of *M. nobiliss* without an added ammonia solution are flagellate (Figure 5A). The sperm of *M. nobiliss* with 1 mmol/L of ammonia solution added showed slight curling at the tail end (Figure 5B); with 2 mmol/L (Figure 5C) of ammonia solution added, moderate curling was observed at the tail end; and with 3 mmol/L (Figure 5D) of ammonia solution added, severe curling at the tail end was evident.

The sperm of *P. fucata martensii* without added ammonia solution are flagellate (Figure 5E). The sperm of *P. fucata martensii* with 1 mmol/L of ammonia solution added (Figure 5F) showed no significant morphological differences compared to those without ammonia solution added; similarly, with 2 mmol/L (Figure 5G) and 3 mmol/L (Figure 5H) of ammonia solution added, no significant morphological differences were observed.

The sperm of *S. mordax* without added ammonia solution are flagellate (Figure 5I). The sperm of *S. mordax* with 1 mmol/L of ammonia solution added showed slight curling at the tail end (Figure 5J); with 2 mmol/L (Figure 5K) of ammonia solution added, moderate curling was evident at the tail end; and with 3 mmol/L (Figure 5L) of ammonia solution added, severe curling at the tail end was observed.

## 4. Discussion

Sperm motility is essential for external fertilization, with high vitality being a key factor enabling sperm to actively seek and approach the egg and penetrate it through the fertilization pore to complete fertilization [36,37]. Evidence suggests that sperm parameters, especially the vitality and motility rate, are crucial for the success of external fertilization [38]. The marine bivalve sperm, upon natural release or artificial extraction, undergo ion exchange in natural seawater under suitable environmental conditions, subsequently becoming activated [39,40]. However, some marine bivalve sperm require chemical substances to trigger activation mechanisms [41]. This study found that the vitality and motility rates of the sperm of *M. nobiliss* were not affected without the addition of ammonia water. Under conditions with 1mmol/L, 2mmol/L, and 3mmol/L ammonia water, the vitality and motility rates of the sperm at different time points were significantly lower than those without ammonia water added. Among them, the addition of 3mmol/L of ammonia water reduced the optimal vitality time and optimal motility rate time of the sperm by 4 h compared to other conditions, indicating that ammonia water has a very significant impact on sperm vitality and motility rates. In conditions without added ammonia water, the vitality and motility rates of *M. nobiliss* sperm were superior to those of *S. mordax* sperm. The addition of ammonia water to both *M. nobiliss* and *S. mordax* sperm led to a more significant decrease in vitality and motility rates with increasing ammonia water concentrations. This could be due to the addition of NH_4_^+^ altering the external pH, inhibiting the sperm vitality and motility rate [42]. External pH is a major factor in inhibiting and inducing the motility of marine bivalve sperm, with alkaline pH conditions potentially inhibiting sperm motility, while neutral and acidic pH can induce sperm motility [23]. It was reported that Japanese Pearl Oyster (*Pinctada fucata martensii* (Dunker, 1872)) sperm, when added to ammonia seawater with a pH of 10.0, showed very limited motility after dilution for 30 s, whereas in seawater with a pH of 8.0~9.0, sperm motility decreased with the increasing NH_4_^+^ ion concentration [43]. Studies have reported the use of NH_4_Cl or NH_3_ to alkalinize the external environmental pH of sea urchins [37,44] and starfish species [45]. Additionally, it has been reported that KOH and NaOH can increase the pH of seawater [46] and can be used to induce and enhance sperm motility. However, the mechanisms underlying the effects of ammonia water on the vitality and motility rates of *M. nobiliss* and *S. mordax* sperm require further investigation. 

The reproductive biology of *P. fucata martensii* exhibits unique characteristics, with sperm collected through artificial dissection remaining inactive in both freshwater and seawater. This suggests that the osmotic pressure and pH alone cannot fully initiate the ion exchange in sperm, thus requiring specific substances to thoroughly activate their ion channels [47]. In production, ammonia seawater with a certain concentration is commonly used to activate sperm [41]. This study found that NH_4_^+^ significantly stimulates the vitality and motility rate of *P. fucata martensii* sperm. Specifically, the vitality and motility rates of *P. fucata martensii* sperm without added ammonia seawater were significantly lower at various times compared to those with ammonia seawater added at different concentrations. Among them, 2mmol/L of ammonia seawater was the most effective in activating *P. fucata martensii* sperm, exhibiting higher sperm vitality and motility rates, and had the best effect on the sperm vitality and motility rate within 0-16 h of activation, consistent with previous research findings [48]. It has been reported that NH_4_^+^ can significantly affect sperm cells and is the only cation that can open ion channels on the membrane of *P. fucata martensii* sperm cells. It can fully open these ion channels on the sperm cell membrane, leading to the exchange of Na^+^ and Cl^−^ [49,50]. Therefore, the presence of NH_4_^+^ can stimulate sperm to enter a fully activated state. Additionally, the presence of NH_4_^+^ can regulate the process of Ca_2_^+^ exchange and Ca_2_^+^ plays a key role in regulating the sperm motility rate [51]. 

The morphology of animal sperm is primarily related to its fertilization method. In mollusks, based on the method of fertilization, sperm are broadly classified into two main categories: primitive type and modified type. Sperm involved in external fertilization are generally of the primitive type, while those involved in internal fertilization are generally of the modified type [50,52]. *M. nobiliss*, *P. fucata martensii*, and *S. mordax*, all of which undergo external fertilization, exhibit sperm morphology that is typical of the primitive structure, consisting of a head, midsection, and tail [53]. This study’s scanning electron microscopy analysis showed that, from the untreated control group to the 3 mmol/L ammonia water treatment group, as the concentration of ammonia water increased, significant morphological changes in the sperm structure were observed. These changes primarily revolved around varying degrees of flagellum curling, which is consistent with previous studies that studied the impact of environmental factors on reproductive cells in marine mollusks [54]. Specifically, in the sperm of *M. nobiliss* and *S. mordax*, increased concentrations of ammonia water led to noticeable morphological variations and an increasing degree of flagellum curling. Sperm exhibited minor morphological changes after treatment with 1mmol/L of ammonia water, and at the highest ammonia water concentration, severe flagellum curling was also observed, which, in turn, affected sperm vitality and motility rates. Changes in the ionic content of water bodies can induce cytotoxic responses [55], and even low levels of environmental toxins can have a negative impact on the reproductive systems of marine mollusks [56,57]. In natural seawater, the concentration of ammonia is typically very low, ranging from about 0.00001 to 0.0005 mmol/L [58]. Compared to the concentrations used in experiments—1 mmol/L, 2 mmol/L, and 3 mmol/L—the experimental concentrations are far higher than those found in natural seawater. The lowest concentration used in the experiment (1 mmol/L) was approximately 1000 times higher than that of the natural levels of ammonia in seawater. These high concentrations are typically used to assess the potential impact of ammonia on marine organisms under extreme conditions. This setup helps to understand the physiological and biochemical responses organisms may experience under high ammonia exposure [59]. It was reported that the sperm of the Eastern Oyster (*Crassostrea virginica*), exposed to red tide algal blooms caused by Karenia brevis in the water where Brevetoxin neurotoxins were present, showed reduced sperm vitality, fertilization success rates, and embryo and larval survival rates [60]. 

The increase in ammonia water concentration did not show significant differences in the morphology of *P. fucata martensii* sperm, which may be because NH_4_^+^, as the only cation capable of opening ion channels on the membrane of *P. fucata martensii* sperm, allows the sperm to absorb NH_4_^+^ from the water body, thereby conducting Na^+^ and Cl- ion exchange to maintain the NH_4_^+^ content in the water body at a balanced state [61]. Studies have indicated that adding different concentrations of ammonia solution to the sperm of abalone (*Haliotis asinina*) has effects on the percentage of egg release and the relationship between sperm morphology and the fertilization rate. It was found that adding a 0.005% ammonia solution to abalone sperm significantly promoted egg release without affecting changes in sperm morphology and fertilization rates [62]. The mechanism of NH_4_^+^’s effect on the morphology of sperm from different marine mollusks requires further exploration. Additionally, it is important to note that while scanning electron microscopy provides detailed images of sperm morphology, its actual impact on fertilization capability needs to be assessed through further fertilization tests.

## 5. Conclusions

In summary, this study demonstrates and emphasizes the distinct reactions of *M. nobilis*, *S. mordax,* and *P. fucata martensii* to ammonium. In *M.nobilis* and *S. mordax*, higher ammonia levels reduce sperm vitality and motility and cause flagella to curl, potentially decreasing motility. For *P. fucata martensii*, the optimal ammonia concentration for assessing sperm vitality and motility is 2 mmol/L with no sperm morphology change. Further research is needed to understand how NH_4_^+^ affects sperm at the cellular and molecular levels and to assess its long-term impacts on the reproduction and population dynamics of marine bivalves.

## Figures and Tables

**Table 1:** Experimental animal data.

Species	Shell Length (mm)	Wet Weight (g)	Gonad Weight (g)
*M. nobiliss* 1	53.86	51.96	13.57
*M. nobiliss* 2	57.64	58.64	12.35
*M. nobiliss* 3	61.31	61.31	13.61
*P. fucata martensii* 1	63.90	27..37	6.7
*P. fucata martensii* 2	59.75	25.84	7.3
*P. fucata martensii* 3	60.59	27.09	8.6
*S. mordax* 1	65.18	84.46	17.53
*S. mordax* 2	62.43	87.63	19.83
*S. mordax* 3	67.22	85.27	22.60

Note: actual measurement data are to be filled in based on experimental results.

**Table 2:** Experimental group configuration.

Species	Concentration (mmol/L)-pH
*M. nobiliss*	0-(7.80~8.20)
*M. nobiliss*	1-(8.47~8.52)
*M. nobiliss*	2-(9.05~9.12)
*M. nobiliss*	3-(9.29~9.63)
*P. fucata martensii*	0-(7.80~8.20)
*P. fucata martensii*	1-(8.47~8.52)
*P. fucata martensii*	2-(9.05~9.12)
*P. fucata martensii*	3-(9.29~9.63)
*S. mordax*	0-(7.80~8.20)
*S. mordax*	1-(8.47~8.52)
*S. mordax*	2-(9.05~9.12)
*S. mordax*	3-(9.29~9.63)

Note: Concentration of ammonia (in mmol/L) and corresponding pH ranges observed for Noble Scallop *M. nobiliss*, Chinese Pearl Oyster *P. fucata martensii*, and Small Rock Oyster *S. mordax*. The concentrations are specified as 0, 1, 2, and 3 mmol/L with associated pH ranges. A concentration of 0 mmol/L indicates the control group with normal seawater pH, while higher values show increasing ammonia levels and their impact on water pH.

## Data Availability

All data are available upon reasonable request.
